# Biocompatibility and histological responses of eggshell membrane for dental implant-guided bone regeneration

**DOI:** 10.25122/jml-2023-0267

**Published:** 2023-07

**Authors:** Horia Opris, Mihaela Baciut, Simion Bran, Cristian Dinu, Daiana Opris, Gabriel Armencea, Florin Onisor, Bogdan Bumbu, Grigore Baciut

**Affiliations:** 1 Maxillofacial Surgery and Implantology, Faculty of Medicine, Iuliu Hatieganu University of Pharmacy and Medicine, Cluj-Napoca, Romania; 2 Department of Oral Surgery, Faculty of Medicine and Pharmacy, University of Oradea, Oradea, Romania

**Keywords:** eggshell membrane, biocompatibility, bone regeneration, rat model

## Abstract

Guided bone regeneration (GBR) utilizing eggshell membrane (ESM) as a potential biomaterial for dental implant therapy augmentation was explored in this study. ESM, an environmentally friendly waste product, possesses collagen-rich characteristics. The biocompatibility and histological responses of ESM were investigated in a rat model. Twelve young adult Wistar rats were used in this study. ESM samples were implanted in subcutaneous and intramuscular pockets, and samples were collected at 48 hours, 4 weeks, and 8 weeks post-implantation. Histological analysis revealed the changes in ESM over time. Results showed that ESM maintained its structural integrity, induced a moderate cellular response, and exhibited slow degradation, indicating potential biocompatibility. However, the lack of organized collagen arrangement in ESM led to the formation of irregular and polymorphic spaces, allowing cell migration. Encapsulation of ESM by newly proliferating collagen fibers and multinucleated giant cells was observed at later time points, indicating a foreign body reaction. Crosslinking might improve its performance as a separation membrane, as it has the potential to resist enzymatic degradation and enhance biomechanical properties. In conclusion, ESM demonstrated biocompatibility, slow degradation, and lack of foreign body reaction. While not suitable as a complete separation membrane due to irregular collagen arrangement, further research involving crosslinking could enhance its properties, making it a viable option for guided bone regeneration applications in dental implant therapy. This study highlights the potential of repurposing waste materials for medical purposes and underscores the importance of controlled collagen structure in biomaterial development.

## INTRODUCTION

Guided bone regeneration is the most clinically utilized and well-documented approach for local augmentation and defect restitution in the jawbone in combination with dental implant therapy [1]. In addition, multiple studies have indicated that the survival rates of implants placed in guided bone regeneration (GBR)-augmented sites are comparable to those implanted in natural bone [2, 3]. In addition, a large cohort clinical investigation found that up to forty percent of implant patients required GBR as part of their implant therapy [2-4]. In accordance with clinical findings, a substantial number of histological studies on various animal models demonstrate the stimulation of bone growth in experimental defects treated with various forms of GBR barrier membranes [5, 6].

The literature has highlighted several primary limitations of resorbable membranes, including, but not limited to, uncontrolled duration of resorption time, hard tissue, or screws to support the membrane and avoid collapse, the potential presence of membrane remnants near bone and future dental implants, and membrane instability which may lead to impaired blood clot formation and healing [7]. As for the non-resorbable GBR membranes, the main disadvantages are the risk of exposure, soft tissue ingrowth, infection of the grafted tissue after exposure, the need for primary fixation, second-stage surgery for removal of the membrane, and an operator-sensitive approach [7, 8].

Eggshell membrane (ESM) is an abundant industrial and domestic waste that is environmentally acceptable, non-toxic, consists of numerous natural proteins, and is modifiable by carbonization and dissolving [9, 10]. It has been utilized in various engineering applications, including capacitors, batteries, solar cells, catalysis, biosensors, cell culture, wound healing, and bone substitutes [11].

*In vivo* investigations in experimental animals in subcutaneous tissue provide useful information on the reactions a material can induce at a histological level [12, 13]. The ideal biomaterial must have a dynamic surface that does not generate histological changes at the implant interface, such as reabsorbing collagen sutures without causing histological alterations [14]. Other qualities required of the resorbable membrane are biocompatibility (membrane should either degrade or integrate into the host tissues), space maintainer (stable to facilitate bone healing), selective occlusiveness (to impede the ingrowth of soft tissues, but allowing oxygen, nutrients, and other substances), easy to manipulate and bioactivation (membranes can have an active role into bone healing, not just a passive one) [15]. Resorption time varies in current commercially available products from 2 months (D, L-lactide-co-glycolide) to 36 months for Poly(L-lactide) membranes [16]. Current GBR protocols consider 4 to 6 months necessary for bone enhancement [17].

The aim of this research was to evaluate the biocompatibility and histologic reaction of the eggshell membrane in a subcutaneous and intramuscular rat model.

## MATERIAL AND METHODS

### Preparation of eggshell membrane

Eggshells from Gallus domesticus were utilized for this study. The outer shell membrane was carefully extracted after removing the egg contents, including the yolk and albumen. The membrane was treated using a 99% alcohol solution to fix and disinfect it. A total of 12 membrane samples, each measuring 1x1 cm, were produced.

### Study design and experimental procedure

Twelve young adult male Wistar rats (body weight: 120–260 g; age: 8–10 weeks) from the Center for Experimental Medicine at Iuliu Hatieganu University of Medicine and Pharmacy of Cluj-Napoca, Romania, were involved in the study. All procedures followed the guidelines outlined in the Guide for the Care and Use of Laboratory Animals by the National Institutes of Health. Every effort was made to reduce animal suffering, use fewer animals, and employ alternative *in vivo* methods. The animals were kept in climate-controlled quarters. This study complied with the ARRIVE guidelines regarding animal studies [13].

After receiving general anesthesia (10% Ketamidor, Richer Pharma AG, Austria, intraperitoneally), the animals were positioned prone on a wooden platform. After shaving, povidone-iodine was used to rinse the implantation area. The shoulder and hip of each animal were dissected with a number 15 blade. After blunt dissection, we placed the membrane on the left side in a subcutaneous pocket at the shoulder level. At hip level, the biceps femoris muscle was exposed through blunt dissection, and a sample was inserted. Nylon sutures (5/0 Dafilon, B. Braun Melsungen AG, Melsungen, Germany) were used for suturing in separate layers to avoid influencing tissue reactions to the membrane.

Individual boxes with food and water *ad libitum* were used to keep the animals. When the experimental periods were over, the animals were put to death (48h, 4 weeks, and 8 weeks) using an overdose of injectable anesthetic. Biopsy samples were obtained and fixed in 10% buffered formaldehyde (Sigma-Aldrich, Steinheim, Germany), which included the membranes and surrounding tissues (with 1 cm safety margins). Following fixation, tissues were successively dried out, cleaned, impregnated, and embedded in Paraplast (Sigma-Aldrich, Steinheim, Germany). We cut and stained sequential 5 µm thickness sections using the Trichrome Goldenhar technique.

### Histological assessment

For the assessment of the structure of the shell membrane, we used a particular method of histological processing in which we aimed to preserve the structure as accurately as possible. The tissue sample with the membrane was fixed in 10% formalin solution for 3 days. After fixation was completed, the pieces were dehydrated with ethyl alcohol, clarified with 1-Butanol, and embedded in paraffin. Sections with a thickness of 5 micrometers were made and stained with the Goldner Trichrome method. Examination of histological sections was performed under an Olympus BX41 (Olympus, Tokyo, Japan) microscope equipped with a digital image capture DSLR camera Olympus E-330.

## RESULTS

### Histopathologic results at 48 hours

The shell membrane was structurally made up of collagen fibers arranged closely together but without rigorous organization. This connective tissue had characteristics of dense, non-oriented connective tissue. Because non-oriented connective tissue does not have a very rigorous arrangement of collagen fibers, it can be easily appreciated that this membrane can be considered a protective membrane but not a separating membrane, as desired in guided bone regeneration. In other words, this membrane has good mechanical strength, but the random arrangement of collagen fibers means that meshes with a rigorous shape and size are not defined between them but are polymorphous in shape and size. In this regard, the shell membrane cannot be considered an efficient separating membrane as desired in guided bone regeneration because cells can pass through the larger meshes between collagen fibers, whereas the separating membrane should not allow this.

**Figure 1:**
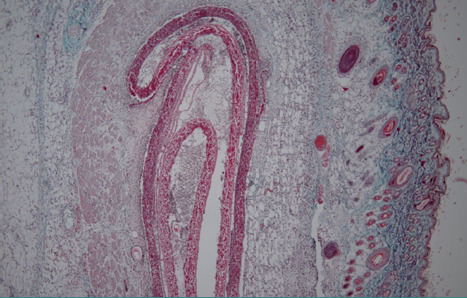
Histologic aspect (20X, Trichrome Masson staining): subcutaneous tissue sample 48 hours after implantation, showing the easily visible shell membrane extending towards the deep muscle

**Figure 2:**
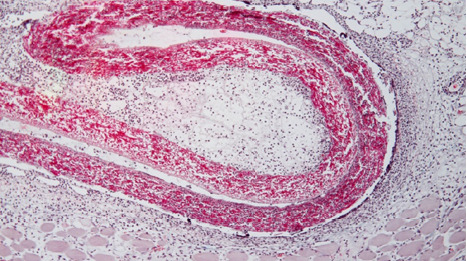
Histologic image showing the dense cellular infiltration in the surrounding tissues of the eggshell membrane, with a noticeable concentration near the shell membrane

In the subcutaneous tissue at the first harvest (48 hours), the shell membrane was readily visible, extending towards the proximity of deep muscle regions (Figure 1). A cellular infiltrate was present in the surrounding tissues and showed densification in the immediate vicinity of the shell membrane (Figure 2).

**Figure 3:**
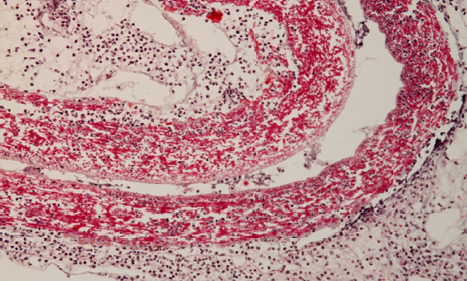
Histologic aspect of an implantation site showing moderate edema and tearing of the shell membrane, causing significant changes in the mesh structure of the membrane fibers

**Figure 4:**
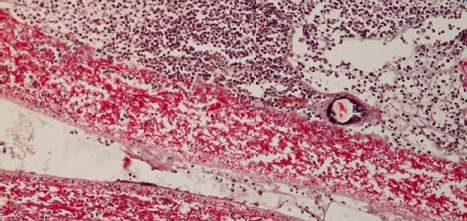
Microscopic image of the eggshell membrane with a highly polymorphic and irregular mesh structure resulting from pressure of edema fluid on the shell membrane fibers at the implantation site

The implantation area of the shell membrane was covered by moderate edema, which also extended to the membrane, resulting in a certain degree of tearing. Consequently, the meshes delimited between the membrane fibers changed due to the pressure of the edema fluid, becoming significantly larger and highly polymorphic both in shape and size (Figures 3 and 4).

**Figure 5:**
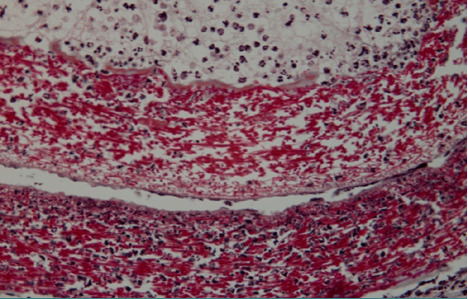
Histologic analysis showing the enlarged gaps between shell membrane fibers, allowing easy passage of cells across the membrane

**Figure 6:**
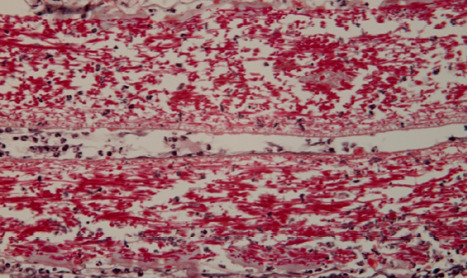
Highly magnified image of the shell membrane fibers, with some gaps large enough to permit multiple cells to pass through at once

The gaps between the membrane fibers became so large that they allowed cells to easily pass from one side of the membrane to the other; even more, cells could pass through the largest gaps at once (Figure 5 and 6).

**Figure 7:**
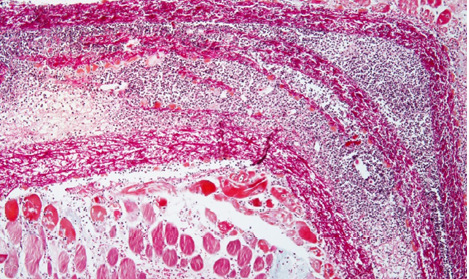
Microscopic image of the eggshell membrane implanted in muscle tissue, surrounded by moderate edema and abundant cellular infiltrate in the immediate vicinity of the membrane, gradually becoming less concentrated as the distance from the membrane increases

Similar trends were observed when the membrane was implanted within muscle tissues, with the surrounding tissues reacting to the presence of the foreign material. The area here was again surrounded by moderate edema and abundant cellular infiltrate in the immediate vicinity of the membrane and became gradually more discrete as one moved away from the membrane (Figure 7).

**Figure 8:**
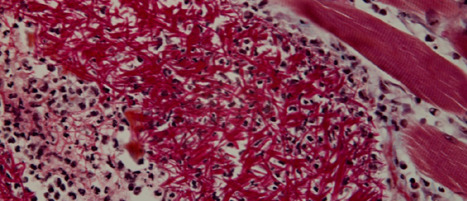
Edema has torn the membrane, enlarging the meshwork bounded by collagen fibers, allowing cells to move from one side to the other

The membrane also appeared torn by edema, with significant enlargement of the meshwork bounded by collagen fibers so that cells could move from one side of the membrane to the other, individually or in groups (Figure 8).

### Histological aspect at 4 weeks

**Figure 9:**
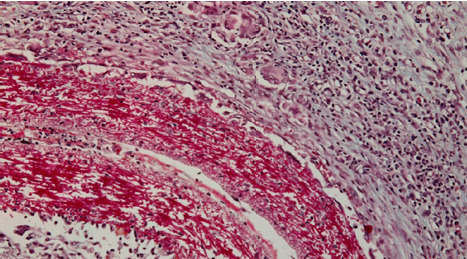
Onset of foreign body granuloma at 8 weeks post-surgery, with preserved interfibrillar edema causing mesh enlargement and loss of separating function in the shell membrane

**Figure 10:**
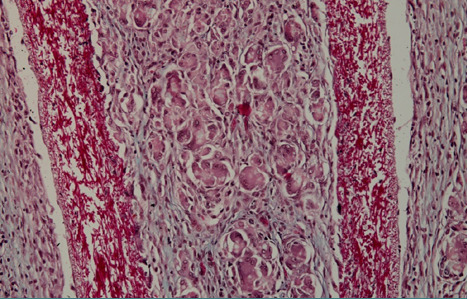
Microscopic image showing newly proliferating collagen fibers and multinucleated giant cells in the connective tissue surrounding the shell membrane, with variation in the number of cells observed between different cases and areas

**Figure 11:**
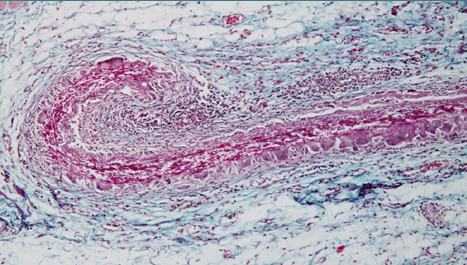
Microscopic image showing complete encapsulation of the shell membrane at 4 weeks post-surgery, with multinucleated giant cells arranged near the membrane and surrounding virtually the entire surface

At 4 weeks postoperatively, the edema decreased in intensity, the cellular infiltrate was reduced, but multinucleated giant cells appeared, which suggests the onset of a granulomatous reaction in the form of a foreign body granuloma. It is noteworthy that at the level of the shell membrane, the interfibrillar edema was mostly preserved so that the enlarged meshes between the collagen fibers of the shell membrane were preserved at large sizes, practically cancelling the function of the separating membrane (Figure 9). The connective tissue around the shell membrane consisted mainly of newly proliferating collagen fibers and multinucleated giant cells, with their frequency varying across cases and areas (Figures 10 and 11).

This connective tissue tended to organize in the form of a thick connective capsule to isolate the shell membrane from surrounding tissues. In other words, there was a process of encapsulation of the shell membrane, which the body perceived as a foreign body. The body recognized the shell membrane as foreign, although this recognition did not trigger severe immunological reactions of rejection. Instead, the body treated it as a foreign entity, triggering a defense mechanism that temporarily encapsulated it to break the connection with its own tissues. This encapsulation gave the organism a longer period to gradually degrade the shell membrane until it was eliminated.

### Histological aspect at 8 weeks

**Figure 12:**
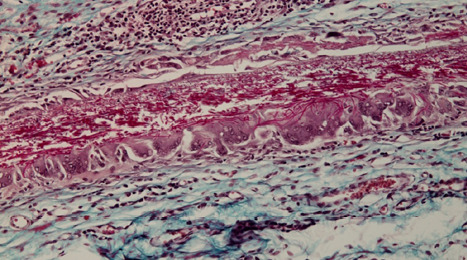
Close-up of the surface of an encapsulated shell membrane at 8 weeks post-surgery, with multinucleated giant cells fused and forming a barrier around the membrane

**Figure 13:**
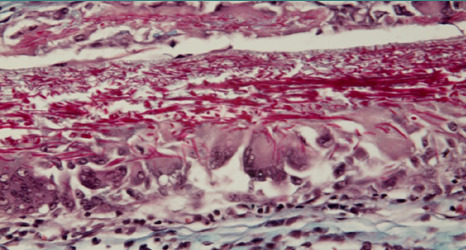
Microscopic image showing enlarged interfibrillar spaces at the level of the shell membrane due to a slow process of fibrinolysis, causing fragmentation of the membrane fibers

At 8 weeks, the shell membrane was totally encapsulated, and multinucleated giant cells fused and appeared arranged near the membrane, which surrounded virtually the entire surface (Figure 12). At the level of the shell membrane, the interfibrillar spaces appeared even larger, and the appearance was because of a slow process of fibrinolysis taking place with fragmentation of the fibers in the membrane structure (Figure 13).

## DISCUSSION

This study aimed to evaluate the histological tissue response of the host to a novel resorbable membrane composed of eggshell membranes. This membrane was made of collagen, which is biodegradable. The arrangement of the fibers offered good mechanical proprieties, but at the cellular level, the mesh allowed for cell migration.

Collagen fiber fragments are gradually removed by phagocytosis by multinucleated giant cells, a highly active process during this experiment phase. However, the actual intracellular degradation of these fiber fragments was a slow process. In this context, the membrane will persist for a period, which, in our opinion, will not be very short, but we cannot say exactly how long. What is certain is that the shell membrane is biodegradable and that degradation is slow, so from this point of view, it would be suitable for use in the body because, as we know, rapid degradation of collagen membranes is disadvantageous. Unfortunately, these qualities are insufficient to conclude that the shell membrane can be used as a separating membrane in guided bone repair. In addition to these qualities, the shell membrane also has disadvantages linked to the fact that the collagen fibers in its structure are not rigorously arranged. As a result, they do not form regular meshes of a suitable and comparable size and subsequently do not totally prevent cell migration through the membrane. The handling of the membrane was relatively easy, and it would be a suitable candidate for clinical use from this standpoint. In addition, the fibers in the structure of the shell membrane were relatively easily dissociated from the body fluids that agglomerate in the area due to the edema triggered by the surgery required to insert the membrane and, to some extent, also by the presence of the membrane as a foreign body. The occlusive nature of the membrane is somewhat limited, allowing smaller cells to traverse its mesh. However, this study lacks the necessary model to assess the extent to which this factor impacts bone healing. Further research is needed to fully evaluate the membrane's capacity to facilitate bone healing within an *in vivo* GBR model.

In comparison, a polylactic acid membrane showed signs of degradation in a study on dogs and rabbits, making it difficult to distinguish it in the tissue. Some inflammatory cells, primarily plasma, and lymphocytes, were visible in the connective tissue next to the membrane [18, 19].

On the other hand, a clinical study used a collagen membrane with a 9–12-month resorption period. Despite this, the exposed portion over the socket is usually resorbed before the 8-week visit. The socket entrance's loss of barrier function had no clinical or histologic effects. Tissue healing and contours were good in this area. Therefore, primary closure over the socket entrance may not be necessary for good clinical and histologic outcomes with this collagen membrane [20].

A paper regarding the use of glutaraldehyde cross-linking on a collagen membrane determined that this membrane began to be absorbed very early, and by day 15, it was fully absorbed [21].

In a different histologic study using the pericardium membrane, the latter could still be clearly seen eight weeks later. Blood vessel density increased, but there were no indications of an inflammatory response. On the surface of the grafts, a connective tissue rich in collagen fibers replaced a natural collagen membrane (Bio-Gide^®^, Geistlich Pharma AG, Wolhusen, Switzerland) that was nearly completely degraded. The pericardium membrane also seemed to have mostly reabsorbed after 12 weeks. On newly formed bone tissue, the periosteum with abundant collagen fibers had replaced it [22].

When evaluating the Bio-Gide^®^ membrane, host cells were able to enter the natural collagen membrane's central regions without degrading it, according to another histologic study. As a result, there was no collapse of the implantation bed caused by material degradation. However, the membrane successfully assimilated into its implantation bed. The implantation bed's metabolism did not significantly differ from the nearby subcutaneous tissue's [23].

A non-cross-linked collagen membrane *in vivo* and *in vitro* study showed that at 20 weeks after implantation, a diverging histological picture of the two membranes becomes apparent. The collagen fibers in the first membrane, made of highly purified non-cross-linked porcine collagen fibers mixed with porcine elastin fibers, continued to be tightly packed with very little interfibrillar space. Blood vessels were being formed. This membrane did not show obvious signs of breakdown and still maintained its integrity. The second membrane (Bio-Gide^®^ membrane), which had a bi-layered structure and non-cross-linked porcine Type I and III collagens, had a noticeable degree of degradation, as seen on histological slides [24].

Cross-linked membranes resisted enzymatic degradation, according to another study. Cross-linked membranes remained intact 20 days postoperatively, while non-cross-linked membranes degraded steadily. Cross-linking improved collagen interactions and structural integrity [25].

Among other methods, physical/chemical crosslinking, ultraviolet radiation, genipin, glutaraldehyde, and 1-ethyl-3-(3-dimethylaminopropyl) carbodiimide hydrochloride (EDC) can improve collagen matrix stability and biomechanical properties. Exogenous crosslinking substances have been found to significantly increase the stability and decrease the antigenicity of collagen-based tissues. The mechanical properties of biological tissues are improved by the formation of extra inter- or intramolecular crosslinks within the collagen fibers [26].

To summarize, the eggshell membrane has multiple qualities that would recommend it, including its relatively prolonged persistence over several months and its gradual biodegradation. Nonetheless, certain drawbacks exist, such as significant alterations upon contact with bodily fluids, evidenced by edema and expansion of interfibrillar spaces. These challenges might potentially be mitigated through cross-linking the membrane. Unfortunately, the primary limitation of the eggshell membrane is its lack of rigorous fiber arrangement, resulting in the formation of polymorphic mesh patterns in terms of size and shape. Consequently, even if the membrane were subject to cross-linking, some of the interfibrillar meshes would still be of a size that would allow cells to pass through the membrane. In this situation, we consider that the shell membrane does not provide a total separation but a partial one, so we do not believe it is suitable as a separating membrane, at least in bone tissue. However, we support its use for alternative purposes in relation to other types of tissues, where it could be used as a protective membrane for a period to promote some repair processes that are somewhat slower but not as slow as bone regeneration.

## CONCLUSION

The eggshell membrane does not determine a foreign body reaction. Our study showed it can be used as an occlusive barrier but not as a separation membrane. It proved to be a potential vehicle for other substances that may enhance its properties. Because it consists of collagen, it is highly biocompatible, resorbable, and biodegradable by the organism.

Crosslinking may enhance the eggshell membrane properties to become a valid guided bone regeneration product, although the polymorphism of the meshes may be an issue. Further research is needed to better understand the processing steps to utilize this cost-effective and readily available biomaterial.
